# More Than Just Statics: Temporal Dynamic Changes in Inter- and Intrahemispheric Functional Connectivity in First-Episode, Drug-Naive Patients With Major Depressive Disorder

**DOI:** 10.3389/fnhum.2022.868135

**Published:** 2022-04-08

**Authors:** Yu Jiang, Yuan Chen, Ruiping Zheng, Bingqian Zhou, Ying Wei, Ankang Gao, Yarui Wei, Shuying Li, Jinxia Guo, Shaoqiang Han, Yong Zhang, Jingliang Cheng

**Affiliations:** ^1^Department of Magnetic Resonance Imaging, The First Affiliated Hospital of Zhengzhou University, Zhengzhou, China; ^2^Key Laboratory for Functional Magnetic Resonance Imaging and Molecular Imaging of Henan Province, Zhengzhou, China; ^3^Engineering Technology Research Center for Detection and Application of Brain Function of Henan Province, Zhengzhou, China; ^4^Engineering Research Center of Medical Imaging Intelligent Diagnosis and Treatment of Henan Province, Zhengzhou, China; ^5^Key Laboratory of Magnetic Resonance and Brain Function of Henan Province, Zhengzhou, China; ^6^Key Laboratory of Brain Function and Cognitive Magnetic Resonance Imaging of Zhengzhou, Zhengzhou, China; ^7^Key Laboratory of Imaging Intelligence Research Medicine of Henan Province, Zhengzhou, China; ^8^Department of Psychiatry, The First Affiliated Hospital of Zhengzhou University, Zhengzhou, China; ^9^GE Healthcare, Beijing, China

**Keywords:** major depressive disorder, resting-state functional magnetic resonance imaging, interhemisphere, intrahemisphere, dynamic functional connectivity, static functional connectivity

## Abstract

Several functional magnetic resonance imaging (fMRI) studies have demonstrated abnormalities in static intra- and interhemispheric functional connectivity among diverse brain regions in patients with major depressive disorder (MDD). However, the dynamic changes in intra- and interhemispheric functional connectivity patterns in patients with MDD remain unclear. Fifty-eight first-episode, drug-naive patients with MDD and 48 age-, sex-, and education level-matched healthy controls (HCs) underwent resting-state fMRI. Whole-brain functional connectivity, analyzed using the functional connectivity density (FCD) approach, was decomposed into ipsilateral and contralateral functional connectivity. We computed the intra- and interhemispheric dynamic FCD (dFCD) using a sliding window analysis to capture the dynamic patterns of functional connectivity. The temporal variability in functional connectivity was quantified as the variance of the dFCD over time. In addition, intra- and interhemispheric static FCD (sFCD) patterns were calculated. Associations between the dFCD variance and sFCD in abnormal brain regions and the severity of depressive symptoms were analyzed. Compared to HCs, patients with MDD showed lower interhemispheric dFCD variability in the inferior/middle frontal gyrus and decreased sFCD in the medial prefrontal cortex/anterior cingulate cortex and posterior cingulate cortex/precuneus in both intra- and interhemispheric comparisons. No significant correlations were found between any abnormal dFCD variance or sFCD at the intra- and interhemispheric levels and the severity of depressive symptoms. Our results suggest intra- and interhemispheric functional connectivity alterations in the dorsolateral prefrontal cortex (DLPFC) and default mode network regions involved in cognition, execution and emotion. Furthermore, our study emphasizes the essential role of altered interhemispheric communication dynamics in the DLPFC in patients with MDD. These findings contribute to our understanding of the pathophysiology of MDD.

## Introduction

Major depressive disorder (MDD), characterized by anhedonia, a persistent negative mood, and cognitive impairments, is a common mental disorder (Lu et al., 2013). It has been reported that MDD is the second leading cause of disability in China (Lu et al., 2021). The high recurrence of MDD and the severe distress experienced by the affected individuals leads to a high risk of suicide, which increases the societal and economic burden of the disease (Nock et al., 2009; Richards, 2011). A better understanding of the pathophysiology of MDD is therefore essential to improve therapeutic outcomes and reduce the social burden.

Functional magnetic resonance imaging (fMRI) provides a new direction for investigating resting-state functional connectivity (FC) and offers effective information for evaluating the integration of functional neural networks *in vivo*. Altered whole-brain FC patterns in MDD have been observed in some resting-state fMRI studies (Zhuo et al., 2017, 2020; Shi et al., 2020), and the FC abnormalities were found to be closely related to cognitive impairments and emotional disorders in patients with MDD (Shi et al., 2020). Moreover, researchers have observed decreased long-range connectivity in the visual cortex, local connectivity patterns in the frontal and temporal cortex in patients with MDD (Zou et al., 2016), and associations between altered local connectivity in MDD before and after antidepressant treatment and the improvements in clinical severity (Wang et al., 2018). According to these fMRI studies, the characteristics of under-connectivity and over-connectivity are effective markers for MDD.

Intra- and interhemispheric interactions play an important role in ensuring fast and efficient computation and information processing in many perceptual and cognitive functions including facial recognition, language understanding, and visuospatial processing (Ringo et al., 1994; Kong et al., 2018). Some fMRI studies have reported high levels of interhemispheric FC in the sensorimotor and auditory cortex in human brain (Biswal et al., 1995; Cordes et al., 2000). Intrahemispheric FC was also reported in earlier studies (Quigley et al., 2003), reflecting the phenomenon of intrahemispheric integration. Some studies have suggested that the left hemisphere may be dominant for cognitive processing, while the right hemisphere may be more important for affective processing (Wittling and Roschmann, 1993; Gainotti, 2012). Some mental disorders, including schizophrenia (Zhu et al., 2018) and autism spectrum disorders (Lee et al., 2016), have been related to altered intra- and interhemispheric interactions. Abnormal hemispheric communication patterns have also been observed in patients with MDD (Guo et al., 2018; Ran et al., 2020). Earlier studies have mainly focused on interhemispheric connectivity, namely, voxel-mirrored homotopic connectivity, which is the correlation between each voxel and the corresponding voxel in the opposite hemisphere (Zuo et al., 2010). Using this measure, individuals with MDD have been found to show disruptions in interhemispheric FC, suggesting impairments in interhemispheric communication during cognitive and emotional processing (Guo et al., 2013, 2018; Lai and Wu, 2014; Hermesdorf et al., 2016; Hou et al., 2016). A few resting-state fMRI studies have observed intra- and interhemispheric FC dysfunction in some cognitive and emotional areas in MDD (Jiang et al., 2019; Ran et al., 2020; Ding et al., 2021). These studies indicate that such impairments in intra- and interhemispheric FC are strongly linked to deficits in perceptual, cognitive, and emotional processing in MDD, and that they may thus be a powerful internal brain activity metric that can help our understanding of MDD as well as aid in the discovery of the underlying neural resting-state pathway.

However, these earlier studies have relied on the assumption that functional connections are static during resting-state fMRI scans and have ignored the presence and potential of FC dynamics in patients with MDD. Recent studies on FC have considered the variability in spatial organization of the brain over time, which can be quantified by measuring the temporal variability of neural signals (Zhu et al., 2020; Tang et al., 2022). FC dynamics may be even more evident when mental activity is unconstrained in the resting-state. Some researchers believe that individuals freely engage in some types of mental activity during the conscious resting state (Delamillieure et al., 2010), and that the predominant mental activity influences FC and modular organization throughout the brain (Doucet et al., 2012). Early studies emphasized that dynamic changes in FC may be related to changes in the state of consciousness (Hutchison et al., 2013), in arousal (Chang et al., 2013), in emotional state (Cribben et al., 2012), and in vigilance (Patanaik et al., 2018). The altered temporal characteristics of dynamic FC may be potential biomarkers of various neurological and psychiatric disorders, including Alzheimer’s disease (Jones et al., 2012), schizophrenia (Sakoğlu et al., 2010), and MDD (Cui et al., 2020). By virtue of the significant dynamic FC abnormalities of specific brain regions and networks in MDD which accurately capture changes in neural activity (Zhang et al., 2020; Zhu et al., 2020), characterizing dynamic FC at intra- and interhemispheric levels is necessary to acquire more worthwhile information about changes in brain states and network properties. Altered temporal dynamics in intra- and interhemispheric FC patterns have been reported in autistic children, suggesting that this may be behind the impairments in social communication characteristic of autism spectrum disorders (Guo et al., 2020). To date, the dynamic changes in intra- and interhemispheric FC patterns in MDD remain unclear. Further investigation of FC dynamics may help to offer a novel understanding and explanation of abnormal brain communication in MDD.

Functional connectivity density (FCD) mapping is a data-driven graph theory method that describes the number of connections of each voxel with all other voxels and identifies the distribution of major connected hubs in the whole brain (Tomasi and Volkow, 2010). In comparison with independent component analysis and seed-based FC methods that rely on specific regions or networks, FCD mapping can evaluate the functional properties of the connectomics of the entire brain, thus evaluating the brain’s information communication capability (Zuo et al., 2012). Static FCD (sFCD) using the entire time series reflects the characteristics of FC within whole-brain networks, while dynamic FCD (dFCD) can be utilized to detect abnormalities in brain connectivity over time by integrating the FCD with a sliding window correlation approach (Li et al., 2019). After computing the FCD within each window, the temporal variability in the dFCD could characterize the time-varying FC patterns within the brain networks. To the best of our knowledge, alterations in the dFCD patterns in MDD remain unclear. Since dynamic and static FC patterns have been reported to offer overlapping or complementary information (Qiao et al., 2020), we combined dFCD and sFCD methods to obtain a more comprehensive explanation of the intra- and interhemispheric FC patterns observed in MDD.

In the present study, we thus decomposed the whole-brain FCD into ipsilateral and contralateral parts, which represent intra- and interhemispheric FC, respectively. Similar to global FCD, intra- and interhemispheric FCD can be used to identify the distribution of major connected hubs at hemispheric levels in brain networks. The associations between dFCD variance and sFCD in abnormal brain regions and the severity of depressive symptoms were also analyzed. We hypothesized that patients with MDD would exhibit abnormal dFCD and sFCD patterns of intra- and interhemispheric connections in some brain regions and specific dFCD variance and sFCD changes at intra- and interhemispheric levels related to the severity of their depressive symptoms.

## Materials and Methods

### Participants

A total of 106 individuals were enrolled in this study, including 58 first-episode drug-naive patients with MDD and 48 age-, sex-, and education level-matched healthy controls (HCs). MDD was diagnosed by two trained psychiatrists independently, according to the Structured Clinical Interview of the Diagnostic and Statistical Manual of Mental Disorders (SCID-I/P, Chinese version). To rule out the presence of current or past psychiatric illness, participants were carefully screened through a SCID-I/P. The severity of depressive symptoms was assessed using the 24-items Hamilton Depression Scale (HAMD-24), an auxiliary tool for evaluating clinical status (Hamilton, 1960). Higher HAMD-24 scores correspond to more severe the symptoms of depression. Patients with MDD were experiencing a depressive episode at the time of scanning. The inclusion criteria for patients with MDD were as follows: (1) fulfilled the SCID-I/P diagnostic criteria for MDD; (2) first episode, and never received any antidepressant treatment; (3) a cut-off score ≥ 20 on the HAMD-24; and (4) Han ethnicity and right-handed. HCs were recruited from the local community *via* poster advertisements. The exclusion criteria for all participants included in the current study were as follows: (1) a history of other mental diseases; (2) organic brain disease or serious physical disease; (3) drug abuse or alcohol dependence; and (4) contraindications to MRI examination, such as metal implants or claustrophobia.

Table 1 shows the demographic and clinical characteristics of the MDD and HCs groups. There were no statistical differences between the two groups in terms of age (*t* = −0.950, *P* = 0.345), sex (χ^2^ = 2.045, *P* = 0.153), years of education (*t* = −1.133, *P* = 0.261), or mean framewise displacement (FD) (*t* = −1.728, *P* = 0.088). The range of HAMD-24 scores in the MDD group was 20–69.

**TABLE 1:** Demographics and clinical characteristics of all participants.

Variables	MDD (*n* = 58)	HCs (*n* = 48)	*t*/χ^2^	*P* value
Age (years)	16.55 ± 4.28	17.40 ± 4.77	−0.950	0.345
Sex (male/female)	37/21	24/24	2.045	0.153
Education (years)	10.19 ± 2.54	11.02 ± 4.53	−1.133	0.261
Mean FD	0.13 ± 0.07	0.15 ± 0.10	−1.728	0.088
HAMD-24 (score)	37.36 ± 13.08	NA	−	−

*MDD, major depressive disorder; HCs, healthy controls; Mean FD, mean framewise displacement; HAMD-24, the 24-items Hamilton Depression Scale; NA, not available.*

Written informed consent was obtained from all participants. The study was approved by the Medical Ethics Committee of The First Affiliated Hospital of Zhengzhou University.

### Image Acquisition

MRI data were acquired using a GE Discovery MR750 3.0T scanner (General Electric, Milwaukee, WI, United States), with an eight-channel prototype quadrature birdcage head coil. All participants lay in a supine position and had their head fixed with foam pads to reduce head movement. Earplugs were used to reduce noise stimulation. During scanning, all participants were instructed to keep their eyes closed, stay awake, and avoid intentional thinking. Resting-state fMRI data were obtained using a gradient-echo echo-planar imaging sequence with the following scanning parameters: repetition time/echo time (TR/TE) = 2,000/40 ms, field of view (FOV) = 220 mm × 220 mm, slices = 32, matrix size = 64 × 64, section thickness = 4 mm, gap = 0.5 mm, flip angle (FA) = 90°, and 180 volumes (360 s in total). Structural T1-weighted images were obtained with 3D spoiled gradient echo scan sequence: TR/TE = 8,164/3.18 ms, inversion time = 900 ms, FA = 7°, resolution matrix = 256 × 256, thickness = 1.0 mm, and slices = 188.

### Data Preprocessing

All preprocessing was conducted using the Data Processing and Analysis for Brain Imaging (DPABI)^1^ toolbox. The first 10 volumes were discarded to ensure signal stability, followed by slice timing and realignment. Head movement exceeding 2.5 mm in maximum displacement or 2.5° rotation in angular motion were excluded. FD was computed, and data with mean FD values exceeding 0.5 mm were excluded (Power et al., 2012). Images were normalized to the standard Montreal Neurological Institute (MNI) space, and each voxel was resampled to 3 mm × 3 mm × 3 mm. The normalized images were detrended to correct the linear trend. Then several spurious variances including 24 head motion parameters, cerebrospinal fluid signal and white matter signal were regressed out from the data. The global signal was not regressed, since previous studies have reported that global signal regression may introduce distortions in time series data (Anderson et al., 2011), and that global signal variance and topography can show alterations in mental disorders, including MDD (Yang et al., 2014; Han et al., 2019; Zhang et al., 2019). Furthermore, band pass filtering was carried out at a frequency range of 0.01–0.08 Hz. Finally, scrubbing with cubic spline interpolation was performed to eliminate the influence of head motion and ensure continuous time points. The mean FD was entered as a covariate into the group-level analysis.

### Inter- and Intrahemispheric Functional Connectivity Density Analysis

An sFCD analysis was performed to evaluate global, contralateral, and ipsilateral FC. Automated anatomical labeling (AAL) atlas excluding the cerebellum was used as a cerebral gray matter mask. The data-driven FCD mapping method was applied to calculate the global sFCD, which was defined as the number of connections of each voxel with all other voxels in the brain mask. Then, the global sFCD was decomposed into two parts according to the relative positions of the seed and target voxels: First, into the contralateral (or interhemispheric) sFCD, which refers to the number of FC of a given voxel with all voxels in the opposite hemispheric brain regions, and second, into the ipsilateral (or intrahemispheric) sFCD, which represents the number of FC with all voxels in the same hemispheric brain regions. To evaluate the connectivity between two voxels, linear Pearson correlations were performed, and the correlation threshold was set in accordance with the criterion of *p* < 0.05 (uncorrected) (Zou et al., 2016; Guo et al., 2020; Yang et al., 2021). When the correlation coefficient between two voxels is larger than the correlation threshold, connectivity is considered to be present.

In the dFCD analysis, we applied a sliding window approach to calculate the temporal variability of global, contralateral, and ipsilateral FC. We chose a sliding window ranging from 10 to 180 s, based on a previous study that suggested this size to be appropriate for capturing the dynamic characteristics of intrinsic brain activities (Gonzalez-Castillo et al., 2015). Prior studies have also demonstrated that to avoid introducing spurious fluctuations, the minimum window length should be above 1/*f*_*min*_, where *f*_*min*_ is the minimum frequency of the time series (Leonardi and Van De Ville, 2015). Based on previous research (Guo et al., 2020; Luo et al., 2021; Zheng et al., 2021), a window length of 50 TRs (100s) and a step size of 2 TRs (4s) were selected to compute the temporal variability of FCD. The full-length time course was segmented into 61 windows for each subject. The correlation threshold of dFCD was set to be identical to that in the sFCD analysis (Guo et al., 2020). The global FCD calculated within each window was decomposed into contralateral and ipsilateral FCD, and then the variance of the time-varying FCD patterns over time was calculated to estimate temporal variability in global, contralateral, and ipsilateral dFCD.

### Statistical and Correlation Analysis

For each subject, the dFCD variance and sFCD maps were converted to z-values using Fisher z-transformation to enhance the normality of the data distribution. Then, all normalized images were smoothed with a 6 mm × 6 mm × 6 mm full-width at half-maximum (FWHM) Gaussian kernel. A two-sample *t*-test was employed on the FCD maps to analyze the differences between the MDD and HCs groups. Age, sex, years of education, and mean FD were used as covariates. In order to control the false positive rate, multiple comparisons were corrected using a Gaussian random field theory (GRF) approach at a voxel-wise threshold of *p* < 0.005 with the cluster-level threshold set to *p* < 0.05 (the cluster-level correction refers to family wise error), and cluster-extent threshold was set at 20 voxels.

The brain clusters showing significant global, intra-, and interhemispheric FCD differences between patients with MDD and HCs were defined as regions of interest (ROIs). The values of dFCD variance and sFCD at global and intra- and interhemispheric levels in all ROIs were extracted separately. A Pearson correlation analysis was performed to identify correlations of abnormal dFCD variance and sFCD with depressive symptom severity (*p* < 0.05, uncorrected).

### Validation Analysis

Two additional window lengths (30 and 80 TRs) were applied to test the robustness of the sliding window analysis. Moreover, dFCD variability with 1 TR as the shifting step and 50 TRs as the window length was calculated to estimate the confounding influence of different step sizes on our main dFCD findings.

## Results

### Inter- and Intrahemispheric Dynamic Functional Connectivity Density Variance

**FIGURE 1:**
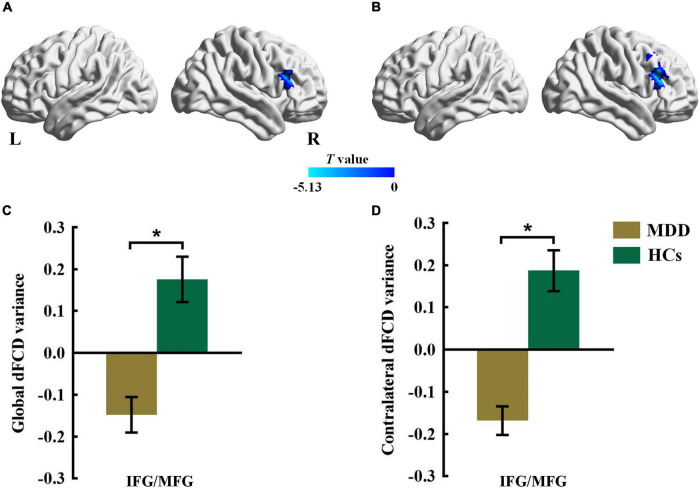
The group difference for **(A)** global and **(B)** contralateral dFCD variance between the MDD and HCs groups. The color bars indicate the *T* value based on two-sample *t*-test. The statistical significance level was set at *P*_*voxel*_ < 0.005, *P*_*cluster*_ < 0.05 under Gaussian random field theory (GRF) corrected. The histogram shows the average **(C)** global and **(D)** contralateral dFCD variance in the IFG/MFG. *p < 0.05. These results are mean ± standard error. Abbreviations: dFCD, dynamic functional connectivity density; IFG, inferior frontal gyrus; MFG, middle frontal gyrus; MDD, major depressive disorder; HCs, healthy controls; L, left; R, right.

Compared with the HCs group, the MDD group showed decreased global dFCD variance in the inferior frontal gyrus (IFG) and the middle frontal gyrus (MFG) (Figure 1 and Table 2). Furthermore, the comparisons of the contralateral dFCD variance maps showed patterns that were similar to the global dFCD variance (Figure 1 and Table 2). The MDD group showed decreased contralateral dFCD variance in the IFG/MFG compared to the HCs group (Figure 1 and Table 2). No significant difference in ipsilateral dFCD variability was found between the MDD and HCs groups.

**TABLE 2:** Altered global and contralateral dFCD variance in MDD group.

	Brain areas	Hemi	Voxels	BA	MNI coordinates			*T* value
					x	y	z	
**Global dFCD variance**		**MDD<HCs**						
Cluster 1	Inferior frontal gyrus	R	115	46	57	36	18	−4.340
	Middle frontal gyrus							
**Contralateral dFCD variance**		**MDD<HCs**						
Cluster 1	Inferior frontal gyrus	R	226	46	51	24	24	−5.131
	Middle frontal gyrus							

*MDD, major depressive disorder; HCs, healthy controls; BA, Brodmann Area; Hemi, hemisphere; dFCD, dynamic functional connectivity density; MNI, montreal neurological institute; R, right.*

### Inter- and Intrahemispheric Static Functional Connectivity Density

**FIGURE 2:**
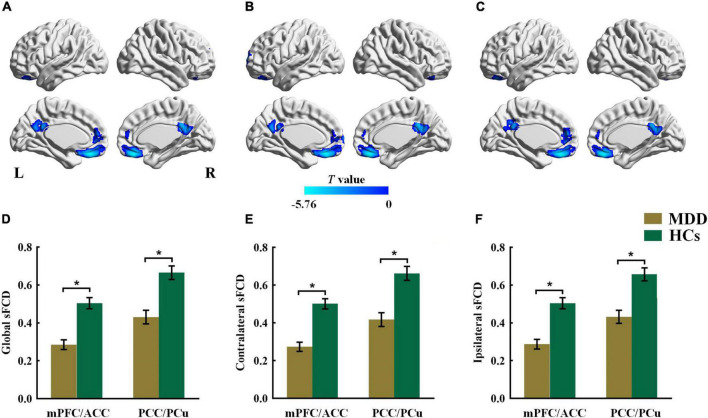
The group difference for **(A)** global, **(B)** contralateral and **(C)** ipsilateral sFCD between the MDD and HCs groups. The color bars indicate the *T* value based on two-sample *t*-test. The statistical significance level was set at *P*_*voxel*_ < 0.005, *P*_*cluster*_ < 0.05 under Gaussian random field theory (GRF) corrected. The histogram shows the average **(D)** global, **(E)** contralateral and **(F)** ipsilateral sFCD values in the mPFC/ACC and PCC/PCu. *p < 0.05. These results are mean ± standard error. Abbreviations: sFCD, static functional connectivity density; mPFC, medial prefrontal cortex; ACC, anterior cingulate cortex; PCC, posterior cingulate cortex; PCu, precuneus; MDD, major depressive disorder; HCs, healthy controls; L, left; R, right.

As shown in Figure 2 and Table 3, patients with MDD showed decreased global sFCD in the anterior default mode network (DMN) regions, including the medial prefrontal cortex (mPFC) and anterior cingulate cortex (ACC), as well as in posterior DMN regions including the posterior cingulate cortex (PCC) and precuneus (PCu). The group differences in the contralateral sFCD and ipsilateral sFCD revealed patterns similar to those of the global sFCD. Both decreased contralateral sFCD and ipsilateral sFCD in the mPFC/ACC and PCC/PCu were found in patients with MDD (Figure 2 and Table 3).

**TABLE 3:** Altered global, contralateral, and ipsilateral sFCD in MDD group.

	Brain areas	Hemi	Voxels	BA	MNI coordinates			*T* value
					x	y	z	
**Global sFCD**		**MDD<HCs**						
Cluster 1	Medial prefrontal cortex	L/R	488	10/11	−3	39	−21	−5.759
	Anterior cingulate cortex	L						
Cluster 2	Posterior cingulate cortex	L/R	254	31	3	−45	27	−4.117
	Precuneus	L/R						
**Contralateral sFCD**		**MDD<HCs**						
Cluster 1	Medial prefrontal cortex	L/R	440	11	−3	36	−21	−5.713
	Anterior cingulate cortex	L						
Cluster 2	Posterior cingulate cortex	L/R	262	23/31	3	−45	27	−4.303
	Precuneus	L/R						
**Ipsilateral sFCD**		**MDD<HCs**						
Cluster 1	Medial prefrontal cortex	L/R	489	10/11	−3	39	−24	−5.755
	Anterior cingulate cortex	L						
Cluster 2	Posterior cingulate cortex	L/R	215	31	12	−51	21	−4.041
	Precuneus	L/R						

*MDD, major depressive disorder; HCs, healthy controls; BA, Brodmann Area; Hemi, hemisphere; sFCD, static functional connectivity density; MNI, montreal neurological institute; L, left; R, right.*

### Correlation Analysis

There were no significant correlations between dFCD variance or sFCD at global, intra-, and interhemispheric levels in abnormal brain regions and the total HAMD-24 scores (*p* > 0.05, uncorrected).

### Validation Analysis

The results of the contralateral dFCD variance analysis were consistent with our main dFCD results. For the global dFCD analysis, the findings in the IFG/MFG regions with a shifting step of 1 TR were successfully repeated, while the results of the two additional window lengths of 30 and 80 TRs were not preserved after GRF correction. The detailed results of the validation analysis are presented in the Supplementary Material.

## Discussion

Since dynamic and static FC patterns have been reported to offer overlapping or complementary information (Qiao et al., 2020), the current study employed both dFCD and sFCD to compare intra- and interhemispheric FC patterns between patients with MDD and HCs. We found that patients with MDD exhibited decreased interhemispheric dFCD variability in the dorsolateral prefrontal cortex (DLPFC) regions, including the IFG/MFG, which is associated with attention, emotion, and cognitive control (Aron et al., 2014; Song et al., 2019). Both intra- and interhemispheric comparisons showed reduced sFCD in regions related to attention focus and cognitive control, namely the DMN regions (mPFC/ACC and PCC/PCu) in patients with MDD.

To the best of our knowledge, the present study is the first to explore the dynamic abnormalities of intra- and interhemispheric FC in the intrinsic neural networks of patients with MDD. Previous studies have focused on static FC abnormalities at intra- and interhemispheric levels in MDD (Jiang et al., 2019; Ran et al., 2020; Ding et al., 2021), and have suggested the significance of exploring intra- and interhemispheric information transfer pathways in patients with MDD. Our results thus expand these previous static FC findings into the realm of dynamic FC, by using the whole-brain dFCD approach at intra- and interhemispheric levels. Notably, our study reveals abnormal FC patterns of time-varying intrinsic brain functional networks in MDD, which is in accordance with prior studies on abnormal interaction dynamics in the DLPFC and other brain regions in patients with MDD (Cui et al., 2020; Zhang et al., 2020). The combination of dynamic and static functional connectome analyses employed here reflects abnormal integration of intra- and interhemispheric brain regions and therefore point the way for future research in patients with MDD.

Patients with MDD showed decreased interhemispheric dFCD variability in the IFG/MFG relative to the HCs. The IFG and MFG are parts of the DLPFC, with the right IFG playing an important role in emotion and cognitive control (Aron et al., 2014). The right MFG is associated with sustained attention, working memory, and cognitive control functions, and may be the node that links the dorsal and ventral attention networks (Koyama et al., 2017; Yüksel et al., 2018; Song et al., 2019). A previous study showed decreased global sFCD in the IFG and MFG in patients with MDD (Zhuo et al., 2020). Another study by Shi et al. (2020) applied an FC strength (FCS) method to measure all the functional connection values of each voxel and demonstrated decreased global static FCS in the IFG in first-episode drug-naive patients with MDD, indicating that static FCS within DLPFC regions changes during the early stages of MDD (Shi et al., 2020). These studies indicate that the IFG and MFG are crucial hubs of MDD in the brain. Our results extend these findings using the dFCD method and offer nuanced information on the temporal changes in the major FC hubs, which may be masked in traditional static studies. Interhemispheric under-connectivity patterns, rather than intrahemispheric under-connectivity, may contribute to global under-connectivity in the MDD group. The invariable interhemispheric dFCD in the IFG/MFG region exhibited in our study further highlights abnormal dynamic functional coordination in MDD, which points to dysfunctions in attention, emotion, and cognitive control.

Our results show both intra- and interhemispheric sFCD alterations in the mPFC/ACC and PCC/PCu in patients with MDD. The mPFC/ACC is centrally located in the anterior DMN regions and is involved in social perception and cognitive control (Adolphs, 2009). A previous resting-state fMRI study showed decreased intra- and interhemispheric static FC in the mPFC and ACC in MDD, which is in line with our findings (Jiang et al., 2019). Decreased interhemispheric static FC between anatomically symmetrical voxels has also been reported in the mPFC (Guo et al., 2013; Wang et al., 2013; Lai and Wu, 2014) and ACC (Lai and Wu, 2014) in MDD. The PCC/PCu, belonging to a core region of the posterior DMN, plays an important role in episodic memory, attention focus, and internally directed cognition (Maddock et al., 2001; Leech and Sharp, 2014). A previous study showed decreased intra- and interhemispheric static FC in the PCC as well as decreased interhemispheric static FC in PCu in MDD (Ran et al., 2020). However, this study investigated intra- and interhemispheric static FC only in the DMN areas and based their analyses on a ROI-wise correlation approach, which most likely led to the differences in findings compared to our study. In addition, reduced interhemispheric static FC in the PCC (Guo et al., 2013, 2018; Wang et al., 2015) and PCu (Guo et al., 2013; Hermesdorf et al., 2016) have been found in MDD in previous studies. Our study demonstrated that there is no overlap between the results from dFCD and sFCD. The results of intra- and interhemispheric sFCD present complementary information that differs from the information provided by dFCD. By measuring sFCD, the present results demonstrate the impact of impairments in intra- and interhemispheric functional coordination and deepen our understanding of FC abnormalities in MDD.

Surprisingly, we did not find significant correlations between abnormal dFCD variance or sFCD and the severity of depressive symptoms in our patients. We hypothesize that this is attributable to the small sample size and narrow scoring range of HAMD-24. This suggests that while dFCD and sFCD can reflect abnormal intra- and interhemispheric functional connections, they may not be useful as biological indicators for the quantitative analysis of depression severity.

The present study has some limitations. First, while we selected 50 TRs as the window length for the main dFCD analysis to capture the dynamic patterns of intrinsic brain activities (Guo et al., 2020; Luo et al., 2021; Zheng et al., 2021), the optimal window length remains controversial, and the effects of different window lengths are unknown. Second, the sample size of our study is relatively small, which may restrict the generalizability of our findings. Third, future longitudinal investigations are necessary to further verify the findings of this cross-sectional design study. Fourth, our study included teenagers and adults, and the influence of developmental factors on the experimental results should be considered. Future research should focus on age-related changes in inter- and intrahemispheric FC in MDD. Fifth, the duration of the scan from which resting-state fMRI data was derived was 6 min, which is a relatively short period and may give rise to less reliable results for FC analysis (Birn et al., 2013).

## Conclusion

The results of our dFCD analysis present complementary information that differs from the information that sFCD provides at the intra- and interhemispheric level. The present study demonstrates altered interhemispheric dFCD variability in IFG/MFG in patients with MDD. Additionally, we found global alterations in sFCD in DMN regions, including the mPFC/ACC and the PCC/PCu in our patients with MDD. Our findings reveal the impact that impairments in intra- and interhemispheric functional coordination have in MDD, which adds to our understanding of the pathophysiology of this disease.

## Data Availability Statement

The original contributions presented in the study are included in the article/Supplementary Material, further inquiries can be directed to the corresponding author/s.

## Ethics Statement

The studies involving human participants were reviewed and approved by the Medical Ethics Committee of The First Affiliated Hospital of Zhengzhou University. Written informed consent to participate in this study was provided by the participants’ legal guardian/next of kin.

## Author Contributions

YJ, YC, and SH conceived and designed the study. YJ, RZ, and BZ supervised the conduct of the study. YC, RZ, BZ, SL, YW, and AG are responsible for the data acquisition. YJ and YC drafted the initial manuscript, analyzed the data, and took responsibility for the manuscript. YJ, YC, BZ, and YRW assisted with the literature review. JC, YZ, SH, and JG reviewed and revised the manuscript. All authors contributed to the article and approved the submitted version.

## Conflict of Interest

JG is an employee of GE Healthcare. The remaining authors declare that the research was conducted in the absence of any commercial or financial relationships that could be construed as a potential conflict of interest.

## Publisher’s Note

All claims expressed in this article are solely those of the authors and do not necessarily represent those of their affiliated organizations, or those of the publisher, the editors and the reviewers. Any product that may be evaluated in this article, or claim that may be made by its manufacturer, is not guaranteed or endorsed by the publisher.

## Footnotes

1
http://rfmri.org/DPABI
