# Supplementary material for: More Than Just Statics: Temporal Dynamic Changes in Inter- and Intrahemispheric Functional Connectivity in First-Episode, Drug-Naive Patients With Major Depressive Disorder
Source: Front Hum Neurosci. 2022 Apr 8;16:868135. doi: 10.3389/fnhum.2022.868135 (PMC9024080; doi:10.3389/fnhum.2022.868135)
Supplement: Supplementary file 1 [file Data_Sheet_1.docx]

**More than just statics: temporal dynamic changes of inter- and intrahemispheric functional connectivity in first-episode, drug-native patients with major depressive disorder**

**Supplementary materials**

**Supplementary Table 1**

TABLE 1 Altered contralateral dFCD in MDD group (two additional sliding-window lengths)

| Window  lengths |  | Brain areas | Hemi | Voxels | BA | MNI coordinates | | | *T* value |
| --- | --- | --- | --- | --- | --- | --- | --- | --- | --- |
|  |  |  |  |  |  | x | y | z |  |
| 30TRs | Contralateral dFCD | | MDD＜HCs | | | | | | |
|  | Cluster 1 | Inferior frontal gyrus | R | 188 | 46 | 54 | 24 | 24 | -4.936 |
|  |  | Middle frontal gyrus |  |  |  |  |  |  |  |
| 80TRs | Contralateral dFCD | | MDD＜HCs | | | | | | |
|  | Cluster 1 | Inferior frontal gyrus | R | 161 | 46 | 57 | 33 | 21 | -4.901 |
|  |  | Middle frontal gyrus |  |  |  |  |  |  |  |

Abbreviations: MDD, major depressive disorder; BA, Brodmann Area; Hemi, hemisphere; dFCD, dynamic functional connectivity density; MNI, montreal neurological institute; L, left; R, right; HCs, healthy controls.

**Supplementary Table 2**

TABLE 2 Altered global and contralateral dFCD in MDD group (shifting step = 1TR)

|  | Brain areas | Hemi | Voxels | BA | MNI coordinates | | | *T* value |
| --- | --- | --- | --- | --- | --- | --- | --- | --- |
|  |  |  |  |  | x | y | z |  |
| Global dFCD | | MDD＜HCs | | | | | | |
| Cluster 1 | Inferior frontal gyrus | R | 115 | 46 | 57 | 36 | 18 | -4.340 |
|  | Middle frontal gyrus |  |  |  |  |  |  |  |
| Contralateral dFCD | | MDD＜HCs | | | | | | |
| Cluster 1 | Inferior frontal gyrus | R | 226 | 46/45 | 51 | 24 | 24 | -5.131 |
|  | Middle frontal gyrus |  |  |  |  |  |  |  |

Abbreviations: MDD, major depressive disorder; BA, Brodmann Area; Hemi, hemisphere; dFCD, dynamic functional connectivity density; MNI, montreal neurological institute; L, left; R, right; HCs, healthy controls.

**Supplementary Figure 1**


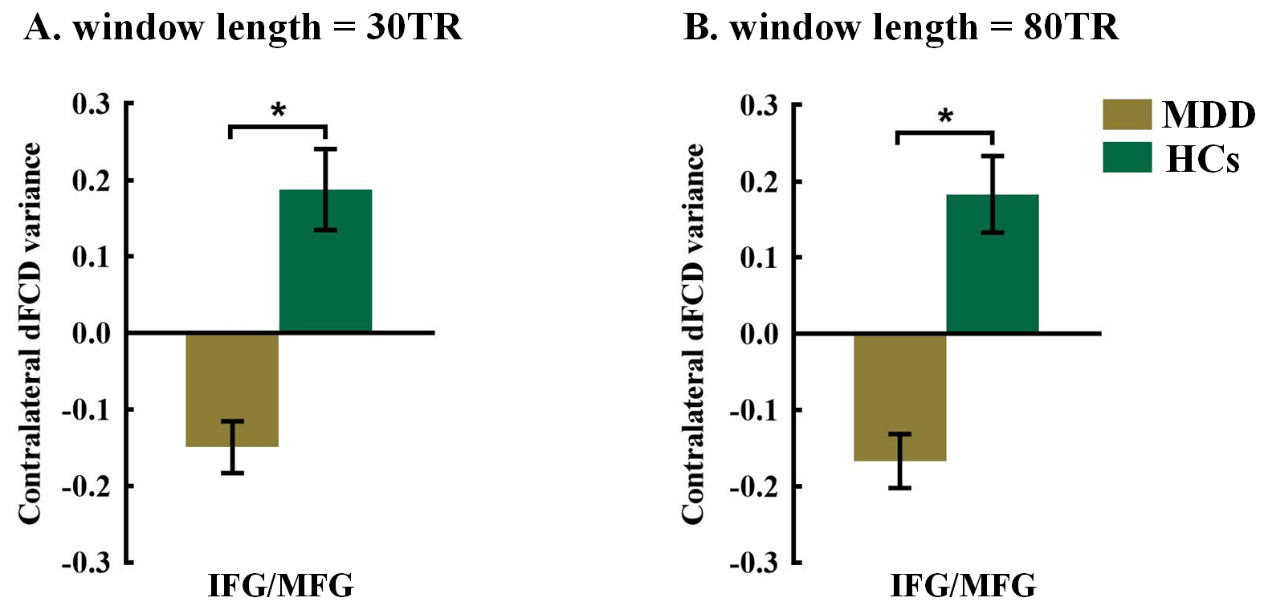


**Supplementary Figure 1. Validation analyses in two additional sliding-window lengths**. Different sliding-window lengths were chosen to verify the robustness of our findings. A. the two-sample *t*-tests analyses results in 30TR (60 s) sliding-window lengths. B. the two-sample *t*-tests analyses results in 80TR (160 s) sliding-window lengths. The statistical significance level was set *P*_voxel_ < 0.005, *P*_cluster_ < 0.05 under Gaussian random field theory (GRF) correction. dFCD, dynamic functional connectivity density; HCs, healthy controls; IFG, inferior frontal gyrus; MDD, major depressive disorder; MFG, middle frontal gyrus

**Supplementary Figure 2**


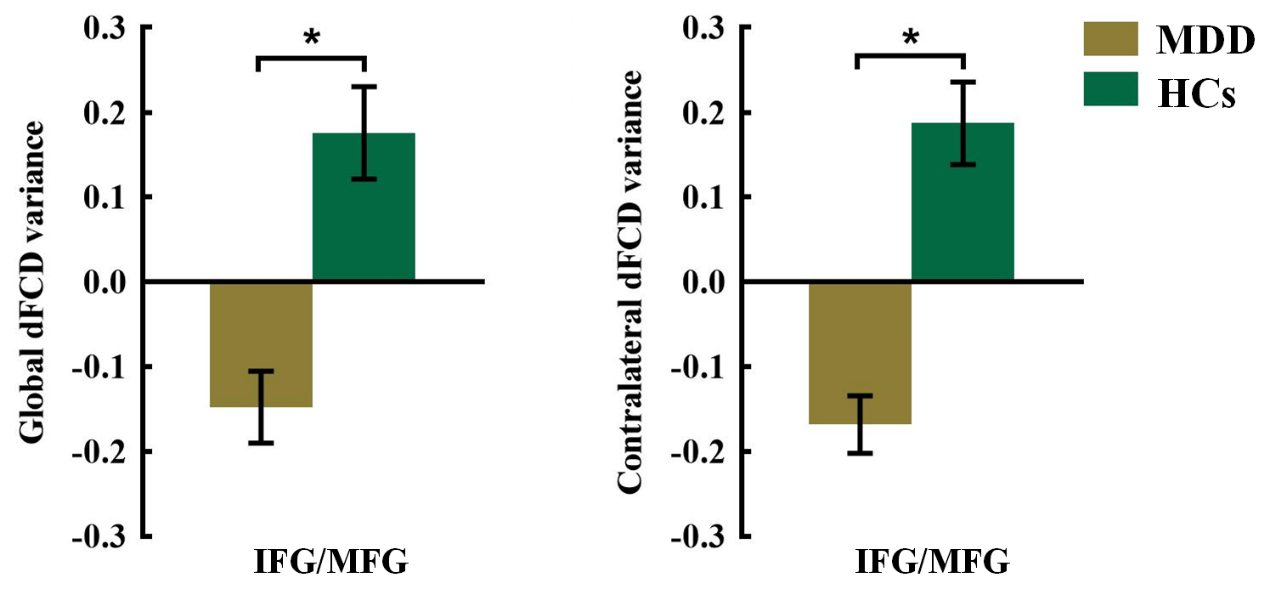


**Supplementary Figure 2. Reproducibility analyses in different shifting step.** An additional shifting step was chosen with 1TR (2 s). The statistical significance level was set *P*_voxel_ < 0.005, *P*_cluster_ < 0.05 under Gaussian random field theory (GRF) correction. dFCD, dynamic functional connectivity density; HCs, healthy controls; IFG, inferior frontal gyrus; MDD, major depressive disorder; MFG, middle frontal gyrus
